# Evaluation of Typical Volatile Organic Compounds Levels in New Vehicles under Static and Driving Conditions

**DOI:** 10.3390/ijerph19127048

**Published:** 2022-06-09

**Authors:** Ruihua Guo, Xiaofeng Zhu, Zuogang Zhu, Jianhai Sun, Yongzhen Li, Wencheng Hu, Shichuan Tang

**Affiliations:** 1 Institute of Urban Safety and Environmental Science, Beijing Academy of Science and Technology, Beijing 100054, China; guoruihua1988@163.com (R.G.); zxf_402@163.com (X.Z.); zzg20022000@163.com (Z.Z.); hwc@bmilp.com (W.H.); 2 State Key Laboratory of Transducer Technology, Aerospace Information Research Institute, Chinese Academy of Sciences, Beijing 100194, China; sunjh@aircas.ac.cn

**Keywords:** vehicle interior air quality, volatile organic compounds, new vehicles, static conditions, driving conditions

## Abstract

In modern societies, the air quality in vehicles has received extensive attention because a lot of time is spent within the indoor air compartment of vehicles. In order to further understand the level of air quality under different conditions in new vehicles, the vehicle interior air quality (VIAQ) in new vehicles with three different brands was investigated under static and driving conditions, respectively. Air sampling and analysis are conducted under the requirement of HJ/T 400-2007. Static vehicle tests demonstrate that with the increasing of vehicle interior air temperature in sunshine conditions, a higher concentration and different types of volatile organic compounds (VOCs) release from the interior materials than that in the environment test chamber, including alkanes, alcohols, ketones, benzenes, alkenes, aldehydes, esters and naphthalene. Driving vehicle tests demonstrate that the concentration of VOCs and total VOCs (TVOC) inside vehicles exposed to high temperatures will be reduced to the same level as that in the environment test chamber after a period of driving. The air pollutants mainly include alkanes and aromatic hydrocarbons. However, the change trends of VOCs and TVOC vary under different conditions according to various kinds of factors, such as vehicle model, driving speed, air exchange rate, temperature, and types of substance with different boiling points inside the vehicles.

## 1. Introduction

Social concerns over indoor-air quality extend not only to the indoor-air environment of newly built apartment houses but also to that of vehicles [1,2]. In modern societies, as a result of urban sprawl, a vehicle cabin has been recognized as a part of the living environment because people are spending increased time in vehicles than ever before during business, shopping, recreation or travel activities [3,4,5,6]. Unfortunately, harmful volatile organic compounds (VOCs), such as benzene, toluene, ethylbenzene, xylenes, styrene, butyl acetate, and undecane, etc., exist in vehicular cabins, which deteriorate vehicle interior air quality (VIAQ) and threaten the health of drivers/passengers [7,8,9,10,11,12,13,14].

The vehicle interior pollution results from the emission of interior furnishing materials and the infiltration of engine exhausts and other exterior environmental pollutants [15,16,17]. In a vehicle cabin, the concentration of VOCs may be higher in comparison to concentrations found in public or private buildings [18,19,20], which may vary in time and are dependent on the interior temperature, humidity, ventilation, vehicle age and other parameters [21,22,23]. Wensing [24] proved that the concentrations of in-vehicle total volatile organic compounds (TVOC) decrease exponentially over a 40-day period from 35~120 mg/m^3^ to 10~30 mg/m^3^. Yoshida and Matsunaga [25] also demonstrated that the TVOC concentration decreased from over 10 mg/m^3^ to 200 μg/m^3^ during the first three years after delivery. The investigation carried out by Grabbs also found that TVOC levels inside new vehicles decreased by more than 90% during a three-week test period [26]. The concentrations of most VOCs declined over time, but increased with increasing interior temperature [27]. Increasing temperature encourages higher desorption of VOCs from interior materials, and the amount of VOCs in vehicles is higher during the summer than during the winter [28]. During vehicle operation, air pollutants originating from interior materials are reduced as more and more VOCs initially captured from interior materials are eventually removed by ventilation [28]. However, heavy traffic problems result in poor air quality in the city, and subsequently cause more serious in-vehicle air pollution problems [29]. Vehicle interior benzene concentrations range from 10~20 μg/m^3^ during freeway travel to 150 μg/m^3^ in heavy urban traffic [30]. VOCs in new vehicles are usually measured under static conditions, in which the air exchange rate is very low (1~3 h^−1^) [31]. However, under other operating conditions, such as setting the fan to fresh air (closing windows), the air exchange rate in the vehicle will increase (13.3~26.1 h^−1^), thus reducing the level of air pollutants in the vehicle [27].

In this study, in order to further understand the level of air quality under different conditions in new vehicles, the concentrations and types of typical VOCs are measured and identified under the static (parked in environment test chamber and in sunshine, vehicle’s engine is off) and driving conditions, respectively. Additionally, the limits specified in Chinese national guidelines for air quality assessment of passenger cars (GB/T 27630-2011) [32] are cited for comparison with the concentrations of typical VOCs.

## 2. Experimental Methods

### 2.1. Vehicicles under Study and Their Pretreatment

The vehicles under study included three brands (Brand A, B, C) of vehicles from different manufacturers, which were all newly domestically produced vehicles in China (tested less than 28 ± five days from their date of production). There were three different models in vehicles of Brand A (A1, A2, A3) and B (B1, B2, B3) respectively, and one model in vehicles of Brand C (C1). The number (n) of vehicles of each model in Brand A, B and C were one (labeled as A1-1, A2-1, A3-1), two (labeled as B1-1, B1-2, B2-1, B2-2, B3-1, B3-2) and six (labeled as C1-1, C1-2, C1-3, C1-4, C1-5, C1-6) (Table 1). All vehicles were well maintained and in good operating condition. None of the vehicles had fuel leakages or any mechanical problems. The covering films, such as plastic film, on the surfaces of the vehicles’ trim materials were ripped off. The passenger compartments were completely free of cigarette smoke and deodorizers. Details of interior materials of all vehicles are presented in Table 1.

### 2.2. Sampling Process under Static Conditions

Under static conditions, the air exchange between the interior and exterior environment of vehicles was significantly reduced when all windows and doors of vehicles were closed. In the case that the concentrations of air pollutants outside of vehicles were very low, VIAQ mainly depended on the amount of VOCs released from vehicle interior sources. That was, the amount of harmful substances released from vehicle interior trims could reflect the situation of vehicle interior air pollution. In this study, the static state was first chosen as the test state of the vehicles, and the vehicle interior air samples were collected in the environment test chamber and in sunshine condition respectively. Standard sampling and analytical methods were used in this experiment.

#### 2.2.1. Vehicle Test Protocol in Environment Test Chamber

As many factors could affect concentrations of VOCs in vehicles, an environment test chamber with a volume of 100 m^3^ was utilized in this experiment, which could provide stable and accurate control of the required temperature, relative humidity (%RH), and airflow velocity, according to set parameters. The air in the chamber was purified by activated carbon filters to reduce the influence of background VOCs on VIAQ. The interior surface of the chamber was constructed with stainless steel, which could minimize adsorption and emission of VOCs [29].

The test protocols utilized with the vehicles (Brand A~C) when sampling the interior air were as follows [33]: (a) The vehicle was moved (the engine was off) to the environment test chamber, and then the chamber’s door was closed. (b) The environmental conditions in the chamber were adjusted according to the set parameters and kept for the whole test duration: environment temperature: 25.0 ± 1.0 °C; relative humidity: 50% ± 10%; airflow velocity: ≤0.3 m/s; background toluene and formaldehyde concentration: ≤0.02 mg/m^3^; background TVOC concentration: ≤0.1 mg/m^3^. The vehicle was aired by opening the windows and doors for 8 h to make a good mixture inside and outside of the vehicle, and then the vehicle was left closed for 16 h, assuming it could reach a steady state pollutant concentration in this period.

The sampling position inside the vehicle was set at head height in the middle of the front two headrests to simulate the height of the driver’s breathing zone. Teflon tubing was used as the sampling line, which was led outside the vehicle from the upper corner of the vehicle’s door, and the length between the sampling device and the sampling location was 2 m. In this way, the sampling process could be done outside the vehicle, thus eliminating the influence of the operator activities on the testing results. In-chamber air samples as background samples were also collected simultaneously. The sampling position in the chamber was within a range of 0.5 m from the test vehicle and at the same height with that in the vehicle. During the whole test procedure, temperature and relative humidity in the chamber were recorded to satisfy the required parameters [29,33].

#### 2.2.2. Vehicle Test Protocol in Sunshine Condition

After the test in the environment test chamber, the vehicles of Brand A were moved outside of the chamber and parked under direct sunlight between 10:00 a.m. and 3:00 p.m. on a sunny and windless day in summer. The ambient temperature during the process of sun exposure should be in the range of 35~37 °C, which could make in-vehicle air temperature increase quickly. In the course of experiments, the Telfon tubing and sensor probe of the thermometer and hygrometer were fixed at the predetermined sampling point (Section 2.2.1), which could auto-monitor and record the temperature and relative humidity inside the vehicles. The tested vehicles were left closed for 4 h (A1-1, A2-1, A3-1) and then the samples of the vehicle’s interior air were collected. The background concentrations of toluene, formaldehyde and TVOC in ambient air should meet the same requirements as in the environment test chamber (Section 2.2.1). Air samples outside the vehicles were collected for blank analysis simultaneously, and the sampling position was within a range of 0.5 m from the test vehicle and at the same height with that in the vehicle.

### 2.3. Sampling Process under Driving Conditions

After the test in the environment test chamber, the vehicles of Brand B and C were moved to the outside of the chamber and were tested on the vehicle proving ground according to the driving test conditions shown in Table 1. Similarly, a sunny and windless day in summer was chosen as the sampling day, and the background concentrations of toluene, formaldehyde and TVOC in ambient air should meet the same requirements as those in the environment test chamber (Section 2.2.1). The windows and vents of the vehicles were kept closed for the entire test procedure. The air conditioner in the vehicle was turned on and set to internal circulation, and the temperature was adjusted to 25 °C. Under driving conditions, since the in-vehicle air samples couldn’t be collected outside the vehicle, it was necessary to bring the sampling device into the vehicle for sampling. The sampling points were arranged strictly according to the requirements of that in static conditions. The sampling workers entering the vehicle had to wear masks and foot covers to reduce the influence of the external air pollutants on the air quality of the tested vehicles. The sampling time began when the driver and sampling workers entered the tested vehicle and turned on the air conditioner in the vehicle (recorded as 0 min). The work from entering the vehicle to when sampling started should be completed within 1 min. The in-vehicle air samples of 0~30 min and 60~90 min were collected with a sampling duration of 30 min. The ambient air samples on the vehicle proving ground as background samples were also collected simultaneously, and the sampling position was at the same height as the sampling position in the vehicle during driving.

### 2.4. Air Sampling and Analysis

Air sampling and analysis was conducted under the requirement of HJ/T400-2007 [33]. In-vehicle VOC emissions (C_6_–C_16_) were taken by active sampling using controlled flow pumps at a rate of 100 mL/min for 30 min onto a stainless steel tube packed with Tenax TA. The samples obtained were stored at 4 °C and were protected from light in a refrigerator until analysis. The thermal desorption-gas chromatography/mass spectrometry (TD-GC/MS) was employed for identification and quantification of in-vehicle VOC emissions. The collected VOCs were thermally desorbed at 270 °C for 3 min. The desorbed compounds were cryogenically focused in a cold trap at −30 °C. After focusing, the trap underwent rapid heating to 280 °C to volatilize the compounds into a GC capillary column through a fused-silicaline heated at 250 °C. The desorbed compounds were identified from the mass spectral data by using the US National Institute of Standards and Technology (NIST). The standard curves were produced with the mixed standard solutions, which were composed of only 9 VOCs, such as benzene, toluene, ethylbenzene, p-xylene, m-xylene, o-xylene, styrene, butyl acetate, and undecane. The identifications of these 9 VOCs were confirmed by their respective chromatographic retention time, and their quantifications were based on a multipoint external standard curve. The response factor of toluene was utilized for quantification of other VOCs and TVOC [22,29].

Aldehydes and ketones in the vehicles were sampled by active sampling using controlled flow pumps at a rate of 400 mL/min for 30 min onto an adsorption tube coated with 2,4-dinitrophenylhydrazine (DNPH). The samples obtained were stored at 4 °C and protected from light in a refrigerator until analysis. Adsorbed aldehydes and ketones were extracted using acetonitrile (HPLC grade) and analyzed by high performance liquid chromatography (HPLC) using a UV detector (360 nm). The HPLC analysis was performed using acetonitrile/water elution (60%/40%, *v*/*v*) as a mobile phase at a flow rate of 1.0 mL/min, an injection volume of 25 μL, and a column temperature of 40 °C. The standard curves were produced with the mixed standard solutions, which were composed of 14 aldehydes and the DNPH derivatives of the ketones, such as formaldehyde-DNPH, acetaldehyde-DNPH, acrolein-DNPH, acetone-DNPH, propionaldehyde-DNPH, butenal-DNPH, butanone-DNPH, methacrolein-DNPH, butyraldehyde-DNPH, benzaldehyde-DNPH, valeraldehyde-DNPH, methylbenzaldehyde-DNPH, cyclohexanone-DNPH, and n-hexanal-DNPH. The identifications of these 14 aldehydes and ketones were confirmed by their respective chromatographic retention time, and their quantifications were based on a multipoint external standard curve.

## 3. Results and Discussion

### 3.1. Temperature and Relative Humidity in Vehicles of Brand A

**Figure 1:**
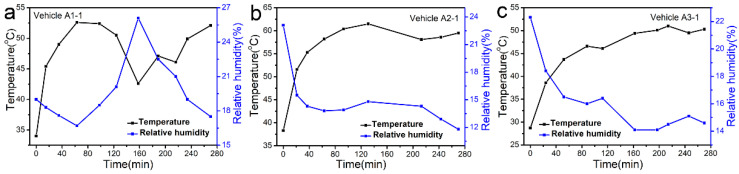
The changes in temperature and relative humidity in sunny conditions inside the vehicles of (**a**) A1-1, (**b**) A2-1 and (**c**) A3-1 during enclosure and air sampling.

The interior temperature and relative humidity in the tested vehicles remained constant in the environment test chamber. however, they were tested in sunny conditions, which would vary with external conditions, such as temperature, whether it was sunny or cloudy out, whether it was windy, etc. Thus, in order to increase the emission of VOCs from vehicle interior trims and reduce the air exchange rate inside and outside the vehicles to the greatest extent, a sunny and windless day in summer, as a “worst case” scenario, was chosen as the sampling day. For a better understanding of the temperature and relative humidity which could be reached inside the vehicles, temperature and relative humidity data inside the tested vehicles were recorded during enclosure and air sampling. As shown in Figure 1, in sunny conditions, the vehicles’ interior air temperature ranged from 28.7 °C to 61.5 °C instead of remaining at 25 °C, and the relative humidity ranged from 11.8% to 26.1% instead of remaining at 50%. The temperature in the vehicle was inversely related to the relative humidity. Without the interference of external conditions, the longer the enclosure time, the higher the temperature in the car.

### 3.2. Interior Concentration changes in Vehicles of Brand A

**Figure 2:**
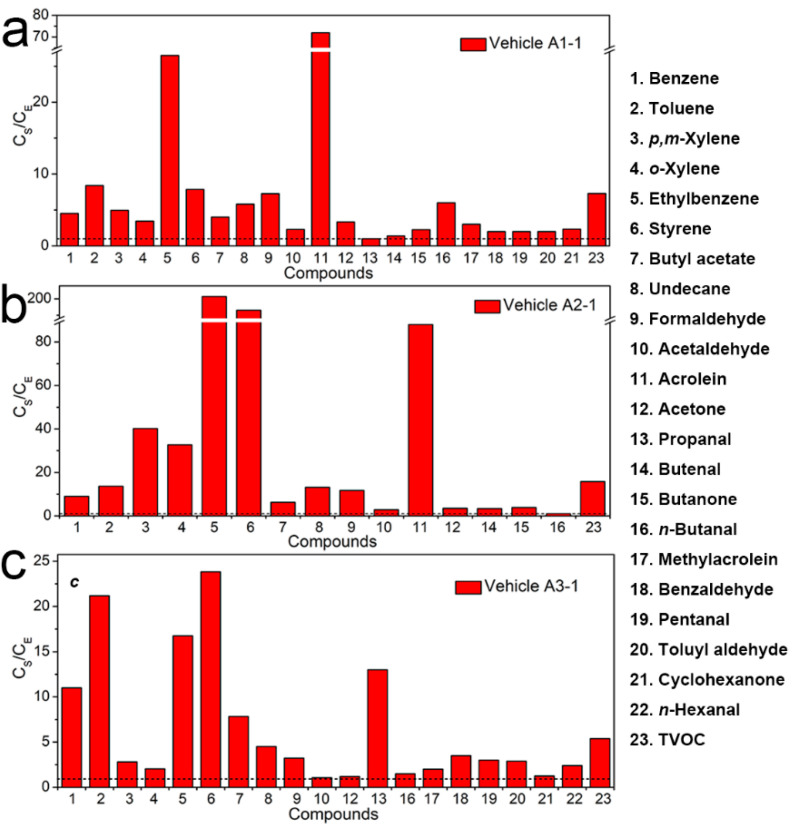
The ratios of concentrations of confirmed compounds and TVOC measured inside the (**a**) Vehicle A1-1, (**b**) Vehicle A2-1, (**c**) Vehicle A3-1 in sunshine condition (C_S_) to that in the environment test chamber (C_E_). The black short dash corresponds to a value of 1.

**Figure 3:**
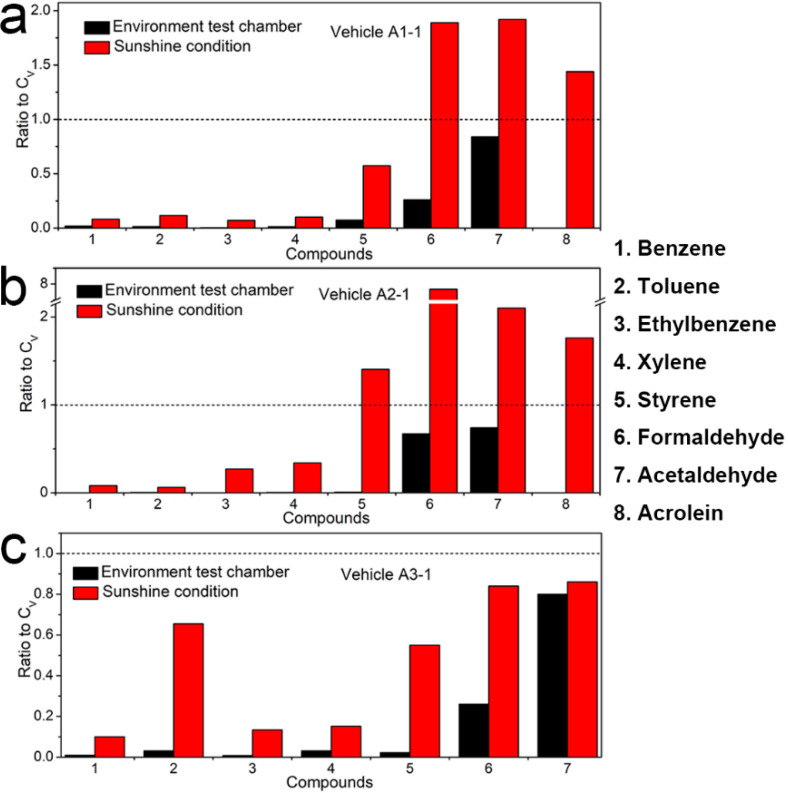
The ratios of concentrations of eight confirmed compounds measured inside (**a**) Vehicle A1-1, (**b**) Vehicle A2-1, (**c**) Vehicle A3-1 in the environment test chamber (C_E_) with sunny outdoor conditions (C_S_) to their corresponding limited values (C_V_) of the national standard GB/T 27630, respectively. The black short dash corresponds to a value of 1.

It had been commonly found that interior temperature was an especially important factor influencing the test results [22,29]. As shown in Figure 2, the ratios (C_S_/C_E_) of concentrations of the confirmed compounds and TVOC measured in Brand A in sunshine condition (C_S_) to that in the environment test chamber (C_E_) were greater than 1, indicating that the VOCs and TVOC pollution concentrations in the three model vehicles increased sharply when the temperature rose from 28.7 °C to 61.5 °C. In addition, as illustrated in Figure 3, when the in-vehicle temperature was 25 °C (in the environment test chamber), the concentrations of the eight in-vehicle confirmed compounds were all lower than their respective limited values in the national standard GB/T 27630-2011. However, when in-vehicle temperature increased, the concentrations of formaldehyde, acetaldehyde, and acrolein in Vehicle A1-1 (Figure 3a) and that of styrene, formaldehyde, acetaldehyde, and acrolein in Vehicle A2-1 (Figure 3b) were 1.89, 1.92, 1.44, 1.40, 7.84, 2.1, and 1.76 times more than their corresponding limited values in the national standard GB/T 27630. Therefore, in-vehicle high temperature was helpful for the evaporation and off-gassing of more VOCs from vehicle interior trims, for the main reason that the release amount of such VOCs as organic solvents, adhesives and additives contained in the interior trim materials could increase more when in-vehicle temperature rose [34,35]. In addition, the reports also showed that the concentrations of VOCs in vehicle interiors increased in concert with the temperature [25,27,36]. Reducing in-vehicle temperature could slow down the VOC emissions from the vehicles’ interior materials.

### 3.3. Interior VOC Type changes in Vehicles of Brand A

Table 2 shows the types of the top 18 VOCs, excluding benzene, toluene, xylene, ethylbenzene, styrene, butyl acetate and undecane, identified in interior air samples of Vehicle A1-1, Vehicle A2-1, Vehicle A3-1, and changes of VOC types under different static conditions. In general, the most alkanes were in the three vehicles under each test condition, but there were more alkanes in the environment test chamber than in the sunshine condition. While in the sunshine condition, there were more other types of compounds, such as alcohols, ketones, benzenes, alkenes, aldehydes, esters, than that in environment test chamber (shown in Table 2). This was because the exposure of the vehicles to direct sunlight in the sunshine condition could lead to interior temperatures up to 61.5 °C, therefore the surface temperatures of the interior materials would be higher, which could cause the volatilization of various chemical substances with different boiling points from the interior surfaces. Thus, the chemical composition and types of VOCs were changed under different static conditions. In conjunction with high temperature, the transmission of solar radiation through glass windows could induce photochemical reactions and the production of degradation of byproducts, which could also cause changes in the chemical composition and types of VOCs.

An environment with a high concentration of VOCs could pose a very large health hazard to drivers and passengers. However, considering that few drivers and passengers would stay in such high-temperature vehicles, it was necessary to further study the changes in the concentrations and types of VOCs in the vehicles under driving conditions when they were exposed to direct sunlight.

### 3.4. Interior Concentration changes in Vehicles of Brand B and C

To further evaluate the differences in VOC concentrations in the vehicles’ interior, air samples were also collected under driving conditions. According to the test results, the concentrations of eight confirmed compounds inside the vehicles of Brand B and C in the environment test chamber were commensurate, and the TVOC concentrations inside Brand C were obviously higher than that of Brand B. In addition, the concentrations of eight confirmed compounds and TVOC inside Brand B and C in the environment test chamber (C_E_) and under driving conditions (C_D(0–30min)_, C_D(60–90min)_) were also compared and discussed.

**Figure 4:**
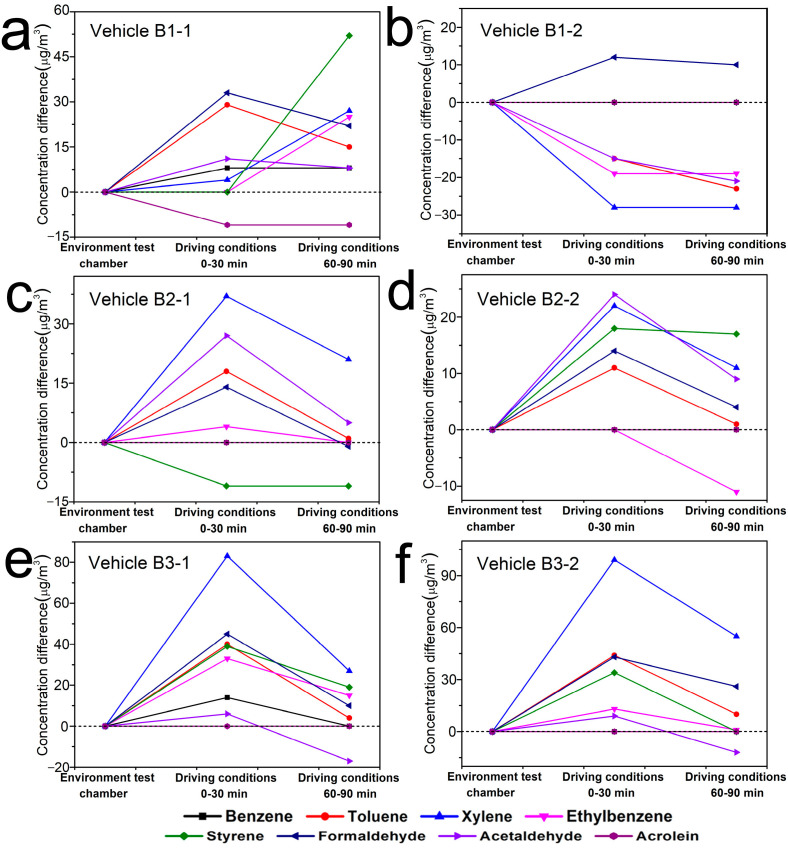
Concentration changes of eight confirmed compounds inside six vehicles of Brand B under different conditions ((**a**). Vehicle B1-1; (**b**). Vehicle B1-2; (**c**). Vehicle B2-1; (**d**). Vehicle B2-2; (**e**). Vehicle B3-1; (**f**). Vehicle B3-2). *Y*-axis indicates the concentration difference between C_D(0–30min)_ and C_E_, C_D(60–90min)_ and C_E_.

**Figure 5:**
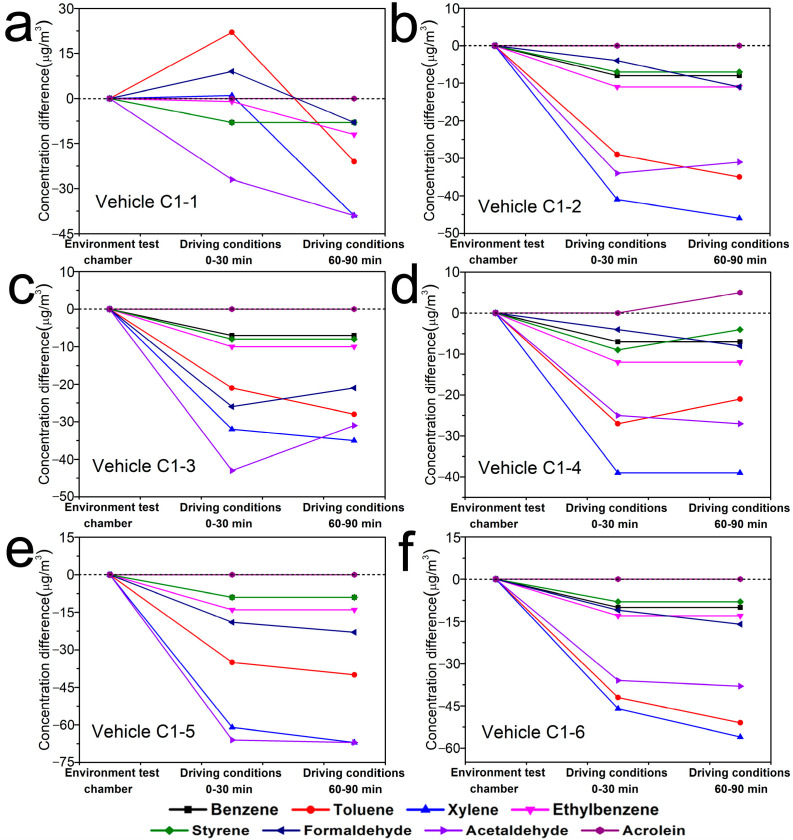
Concentration changes of eight confirmed compounds inside six vehicles of Brand C under different conditions ((**a**). Vehicle C1-1; (**b**). Vehicle C1-2; (**c**). Vehicle C1-3; (**d**). Vehicle C1-4; (**e**). Vehicle C1-5; (**f**). Vehicle C1-6)). *Y*-axis indicates the concentration difference between C_D(0–30min)_ and C_E_, C_D(60–90min)_ and C_E_.

**Figure 6:**
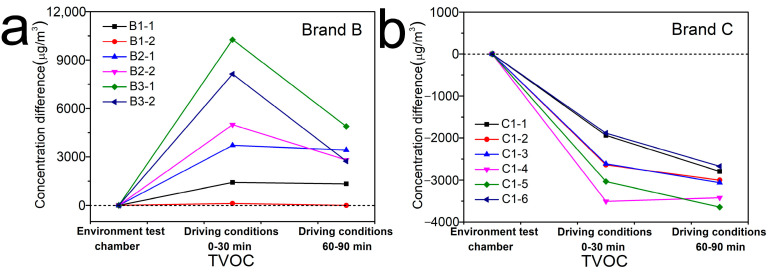
Concentration changes of TVOC inside six vehicles of (**a**) Brand B and (**b**) C under different conditions. *Y*-axis indicates the concentration difference between C_D(0–30min)_ and C_E_, C_D(60–90min)_ and C_E_.

As shown in Figure 4, in general, the concentration changes of eight confirmed compounds inside six vehicles of Brand B were C_D(0–30min)_ > C_D(60–90min)_ ≥ C_E_. But there were some exceptions. Inside the vehicle B1–1, the concentration change of benzene was C_D(0–30min)_ = C_D(60–90min)_ > C_E_, that of xylene was C_D(60–90min)_ > C_D(0–30min)_ > C_E_, that of ethylbenzene and styrene were C_D(60–90min)_ > C_D(0–30min)_ = C_E_, and that of acrolein was C_E_ > C_D(0–30min)_ = C_D(60–90min)_. Inside the vehicle B1-2, the concentration change of formaldehyde was C_D(0–30min)_ > C_D(60–90min)_>C_E_, while that of other compounds were C_E_ > C_D(0–30min)_ ≥ C_D(60–90min)_. Inside the vehicle B2-1, the concentration change of styrene was C_E_ > C_D(0–30min)_ = C_D(60–90min)_, and that of formaldehyde was C_D(0–30min)_ > C_E_ > C_D(60–90min)_. Inside the vehicle B2-2, the concentration change of ethylbenzene was C_E_ = C_D(0–30min)_ > C_D(60–90min)_. Inside the vehicle B3-1 and B3-2, the concentration change of acetaldehyde was C_D(0–30min)_ > C_E_ > C_D(60–90min)_. Whereas, as shown in Figure 5, the change trends of concentrations inside six vehicles of Brand C were clearly different with that inside Brand B, which generally were C_E_ > C_D(0–30min)_ > C_D(60–90min)_. But there were also some exceptions. Inside the vehicle C1-1, the concentration changes of toluene and formaldehyde were C_D(0–30min)_ > C_E_ > C_D(60–90min)_. The concentration changes of acetaldehyde inside vehicle C1-2, that of formaldehyde and acetaldehyde inside C1-3 and that of toluene inside C1-4 were all C_E_ > C_D(60–90min)_ > C_D(0–30min)_. Inside vehicle C1-4, the concentration change of xylene was C_E_ > C_D(0–30min)_ = C_D(60–90min)_, that of acrolein was C_D(60–90min)_> C_E_ = C_D(0–30min)_. Similarly, as shown in Figure 6, the TVOC concentration changes inside the six vehicles of Brand B were all C_D(0–30min)_ > C_D(60–90min)_ > C_E_, while that inside Brand C were C_E_ > C_D(0–30min)_ > C_D(60–90min)_, which had the same trend as that of eight confirmed compounds.

As the air conditioning mode inside the tested vehicles was adjusted to internal circulation, and the infiltration air flow through joints and leaks in vehicle envelopes was the predominant airflow that could affect pollutant transportation inside vehicle cabins [17,37]. Under driving conditions, the vehicles ran at a certain speed, which could accelerate the air exchange inside and outside vehicles. Therefore, the concentration changes of VOCs and TVOC inside the vehicles should be C_E_ > C_D(0–30min)_ > C_D(60–90min)_ in theory, which was consistent with the results tested inside the vehicles of Brand C. But based on the test results of Brand B, except for some substances inside the vehicle B1-1 and B1-2, C_D(0–30min)_ were generally higher than C_E_ and C_D(60–90min)_. That was because the six vehicles of Brand B were all parked and enclosed under direct sunlight (Table 1) before the experiments were carried out under driving conditions, which could accelerate volatilization of VOCs from vehicle interior trims due to the high temperature inside the vehicles. Therefore, it was reasonable that more pollutants were collected inside the vehicles of Brand B at the first 30 min under driving conditions than that in environment test chamber, even though the temperature was the same under these two conditions. However, although the vehicle C1-5 and C1-6 were also parked and enclosed under direct sunlight for 2 h before running on the vehicle proving ground, the C_D(0–30min)_ of VOCs and TVOC were actually lower than their C_E_. That was because different vehicle brands have different air exchange rates. And the higher speed of the vehicles of Brand C than that of Brand B could lead to the increase of the air exchange rate inside and outside Brand C on one hand, while on the other hand, there might be (no air sampling) less pollutants emitted from the interior trims of Brand C than that of Brand B under direct sunlight, and then the concentration of pollutants inside Brand C would decrease rapidly at a higher air exchange rate.

As the vehicles continued to run at a certain speed, the concentration of VOCs and TVOC inside the vehicles would decrease with time due to the air exchange inside and outside the vehicles. Thus, C_D(60–90min)_ of VOCs and TVOC inside Brand B were less than their C_D(0–30min)_. But the air exchange rate value determined whether a longer driving process was required to reduce the concentration of airborne pollutants in the vehicles to be consistent with or lower than that in the environment test chamber. According to the test results shown in Table 3, the C_D(60–90min)_ of eight compounds and TVOC were 1.00~3.27 times and 0.99~7.93 times more than their C_E_, demonstrating that after a period of driving, the concentrations of air pollutants inside the vehicles of Brand B were decreased to the same level with or close to that in the environment test chamber (Figure 4 and Figure 6a). If the air conditioning mode was switched to external circulation (the concentration of ambient air pollutants should meet the requirements in Section 2.2.1), it would take a shorter time to reduce the concentration of air pollutants in the vehicles. The above results further indicated that the concentration values obtained by the standard method (in environment test chamber) were close to the actual exposure level for drivers and passengers.

### 3.5. Interior VOC Type changes in Vehicles of Brand B and C

The difference in the types and boiling points of VOCs inside the vehicles of Brand B and C would also lead to the difference in the trend of TVOC concentration changes inside these vehicles in actual driving conditions. It would take a longer driving time to reduce the TVOC concentration inside these vehicles to a level that was consistent with or lower than that in the environment test chamber.

Table 4 and Table 5 showed types of the top 10 VOCs identified inside the vehicles of Brand B and C, and changes of VOCs types under different test conditions. For the vehicles of Brand B, alkanes were the main pollutants. But for the two vehicles of each model in Brand B, there was no difference in the VOC types under different conditions. Whereas inside the vehicles of Brand C, benzenes and alkanes were the main pollutants. But in the environment test chamber, there were more benzenes and less alkanes than that in driving conditions. In addition to the effect of temperature on the air quality inside the vehicles, air exchange rate inside and outside the vehicles was also the factor influencing the VOCs types.

## 4. Conclusions

The following conclusions can be drawn through the comparison and discussion of the quantitative and qualitative analysis results of VOCs in the vehicle interior air of Brand A~C under different conditions:

(1) The in-vehicle high temperature in the sunshine condition causes higher concentrations and more different types of VOCs released from the interior materials with different boiling points of VOCs than that in environment test chamber. In the vehicles of Brand A, more alkanes were identified in the environment test chamber, while more other types of compounds, such as alcohols, ketones, benzenes, and esters, were identified in the sunshine condition. In the vehicles of Brand C, there are aromatic hydrocarbons and alkanes, and the number of aromatic hydrocarbons is slightly more than that of alkanes. While in Brand B, there are mainly alkanes without aromatic hydrocarbons. Therefore, the drivers and passengers should ventilate their vehicles and turn on the air conditioner to cool down the interior before using a vehicle exposed to the sunshine.

(2) Because of different in-vehicle temperatures, driving speeds, air exchange rates, and types of substances with different boiling points inside the vehicles, the change trends of eight typical organic pollutants and TVOC concentrations in the air of Brand B and C tested vehicles under different conditions are also different, namely:

The vehicles of Brand B: C_D(0–30min)_ > C_D(60–90min)_ ≥ C_E_,

The vehicles of Brand C: C_E_ > C_D(0–30min)_ > C_D(60–90min)_.

The existence of individual exceptions is not excluded.

(3) There are no requirements with regard to the limited values and measurement methods for VIAQ under high temperature in the national standard GB/T 27630 and HJ/T 400. However, according to the results in this study, the concentrations of eight typical organic compounds inside the vehicles exposed to high temperature after a period of driving will be reduced to the same level as or tend to that in the environmental chamber test, indicating that the results of standard test methods are close to the actual driving exposure level. However, due to the variety of pollutants, it takes a longer time for the TVOC concentration to reduce to the same level as that in the environmental chamber test.

## Figures and Tables

**Table 1:** Vehicles under study and corresponding test conditions.

Tested Vehicles			n	Interior Materials			Test Conditions		
							Static Conditions		Driving Conditions
				Seat	Carpet	Interior	Environment Test Chamber	Sunshine Condition	
Brand A	A1	A1-1	1	Leather	Fabric	Plastic	Section 2.2.1	Section 2.2.2	-
	A2	A2-1	1	Fabric					
	A3	A3-1	1	Leather					
Brand B	B1	B1-1	2	Leather	Fabric	Plastic	Section 2.2.1	Closed exposure for 1 h, no sampling	40 km/h
		B1-2							
	B2	B2-1	2					Closed exposure for 1 h, no sampling	40 km/h
		B2-2							
	B3	B3-1	2					Closed exposure for 4 h, no sampling	40 km/h
		B3-2							
Brand C	C1	C1-1	4	Leather	Fabric	Plastic	Section 2.2.1	-	50 km/h
		C1-2							
		C1-3							
		C1-4							
		C1-5	2					Closed exposure for 2 h, no sampling	50 km/h
		C1-6							

**Table 2:** Types of the top 18 VOCs identified in interior air samples of Brand A (excluding benzene, toluene, xylene, ethylbenzene, styrene, butyl acetate and undecane).

Tested Vehicles	Static Conditions	Number of Each Type Compounds							
		Alkanes	Alcohols	Ketones	Benzenes	Alkenes	Aldehydes	Esters	Naphthalene
Vehicle A1-1	Environment test chamber	14	2	1	0	1	0	0	0
	Sunshine condition	8	3	3	2	0	1	1	0
Vehicle A2-1	Environment test chamber	12	1	1	0	0	1	1	2
	Sunshine condition	11	1	2	1	0	0	2	1
Vehicle A3-1	Environment test chamber	14	1	1	1	0	0	0	1
	Sunshine condition	8	2	2	2	1	0	2	1

**Table 3:** The ratios of C_D(60–90min)_ and C_E_ of eight confirmed compounds and TVOC measured inside Brand B.

Compounds	C_D(60–90min)_/C_E_					
	B1-1	B1-2	B2-1	B2-2	B3-1	B3-2
Benzene	/	/	/	/	/	/
Toluene	1.71	/	1.06	1.07	1.21	1.56
Xylene	1.55	/	1.81	1.46	1.77	/
Ethylbenzene	3.27	/	1.00	/	/	1.09
Styrene	/	/	/	2.00	/	/
Formaldehyde	1.73	1.28	0.98	1.09	1.23	2.44
Acetaldehyde	1.4	0.46	1.15	1.30	0.67	0.71
Acrolein	/	/	/	/	/	/
TVOC	5.05	0.99	3.57	3.38	7.93	3.40

“/” means that the value of C_E_ was not detected.

**Table 4:** Types of the top 10 VOCs identified in interior air samples of Brand B.

Tested Vehicles	Test Conditions	Number of Each Type Compounds		
		Alkanes	Alcohols	Alkenes
Vehicle B1-1Vehicle B1-2	Environment test chamber	8	2	0
	Driving condition (0–30 min)			
	Driving condition (60–90 min)			
Vehicle B2-1Vehicle B2-2	Environment test chamber	10	0	0
	Driving condition (0–30 min)			
	Driving condition (60–90 min)			
Vehicle B3-1Vehicle B3-2	Environment test chamber	9	0	1
	Driving condition (0–30 min)			
	Driving condition (60–90 min)			

**Table 5:** Types of the top 10 VOCs identified in interior air samples of Brand C.

Tested Vehicles	Test Conditions	Number of Each Type Compounds					
		Benzenes	Alkanes	Alcohols	Alkenes	Esters	Others
Vehicle C1-1	Environment test chamber	8	2	0	0	0	0
	Driving conditions (0–30 min)	6	4	0	0	0	1
	Driving conditions (60–90 min)	4	6	0	0	0	1
Vehicle C1-2	Environment test chamber	7	2	0	0	0	0
	Driving conditions (0–30 min)	4	4	1	0	0	1
	Driving conditions (60–90 min)	5	5	0	0	0	1
Vehicle C1-3	Environment test chamber	6	2	0	0	1	1
	Driving conditions (0–30 min)	3	2	0	1	0	4
	Driving conditions (60–90 min)	6	4	0	0	0	0
Vehicle C1-4	Environment test chamber	7	2	0	0	1	0
	Driving conditions (0–30 min)	4	6	0	0	0	1
	Driving conditions (60–90 min)	4	5	0	0	0	1
Vehicle C1-5	Environment test chamber	8	2	0	0	0	0
	Driving conditions (0–30 min)	5	5	0	0	0	1
	Driving conditions (60–90 min)	4	6	0	0	0	1
Vehicle C1-6	Environment test chamber	7	2	0	0	0	0
	Driving conditions (0–30 min)	4	5	0	0	0	1
	Driving conditions (60–90 min)	5	5	0	0	0	1

## Nomenclature

VIAQ	vehicle interior air quality
VOCs	volatile organic compounds
TVOC	total volatile organic compounds
C_S_	concentrations of VOCs and TVOC measured inside the vehicles in sunshine condition
C_E_	concentrations of VOCs and TVOC measured inside the vehicles in environment test chamber
C_V_	the limited values of VOCs specified in the standard GB/T 27630-2011
C_D(0–30min)_	concentrations of VOCs and TVOC measured inside the vehicles under driving conditions of 0–30 min
C_D(60–90min)_	concentrations of VOCs and TVOC measured inside the vehicles under driving conditions of 60–90 min
